# Bending Collapse and Energy Absorption of Dual-Phase Lattice Structures

**DOI:** 10.3390/ma17163952

**Published:** 2024-08-09

**Authors:** Zihao Chen, Zijie Zhu, Bangzhen Li, Kehua Leng, Min Yu, Zhixin Huang, Ying Li

**Affiliations:** 1 School of Naval Architecture, Ocean and Energy Power Engineering, Wuhan University of Technology, Wuhan 430064, China; chenzihao1993@gmail.com (Z.C.); huangzhixin1802@163.com (Z.H.);; 2 State Key Laboratory of Explosion Science and Technology, Beijing Institute of Technology, Beijing 100081, China; 3 Beijing Key Laboratory of Lightweight Multi-Functional Composite Materials and Structures, Institute of Advanced Structure Technology, Beijing Institute of Technology, Beijing 100081, China

**Keywords:** dual-phase architecture lattice, energy absorption, bending loading

## Abstract

A dual-phase lattice structure composed of mixed units with hard and soft phase characteristics is proposed in this work. The proposed lattice structure has high specific energy absorption and high compressive strength. The load response and energy absorption characteristics under bending loads were studied through three-point bending tests and numerical analysis methods. The research results indicate that although the deformation modes of the given lattice structure are the same, the dual-phase design strategy significantly improves the bending performance of the lattice structure: the bending modulus is increased by 744.7%, and the specific energy absorption is increased by 243.5%.

## 1. Introduction

Lattice structures have the advantages of being lightweight, with impact resistance, high strength, and high energy absorption efficiency [1,2,3,4,5]. With their unique structure and performance, lattice structures have shown broad application prospects in multiple fields, such as aerospace and shipbuilding [6,7,8,9,10]. In recent years, researchers have conducted extensive research on the mechanical properties of lattice structures and proposed various methods to improve their structural performance [11,12,13,14,15,16,17,18].

Optimizing the design of nodes and columns in lattice structural elements is a very effective method to improve performance. Yan et al. [11] replaced traditional straight columns with non-straight columns with spring characteristics to form a new lattice structure with J-shaped stress–strain responses, achieving nonlinear anisotropic mechanical properties of defect-insensitive biological tissues. Moestopo et al. [19] designed a novel lattice structure with double helix structure characteristics by connecting components with woven nodes. Their structure abandons traditional fixed nodes, significantly improves the elastic deformation and crack propagation suppression ability of the structure, and enhances the fracture toughness and large deformation ability of the structure. Peng et al. [20] adopted a new hybrid design scheme of nonlinear and linear pillars, which achieved a reduction in peak stress and an increase in platform stress. This scheme significantly improves the energy absorption performance of traditional tension-dominated lattice structures by combining the performance advantages of tension-dominated and bending-dominated lattice units.

The multi-level design and gradient design of lattice structures are two other structural optimization design methods. The inspiration for the multi-level design comes from natural multi-level biomaterials such as bones, tendons, and crustaceans in nature [21], which achieve the fusion of multiple structural functions and have good multifunctional characteristics. Meza et al. [12] found that the ratio of strength, stiffness, and relative density of the designed multi-layer nanoscale lattice structure was close to the theoretical limit; moreover, its performance was found to be better than the existing nanoscale lattice structure, which was determined by a study of the multi-level design of the truss structure. In addition, some samples exhibit strong elastic recovery characteristics after compression experiments. Zheng et al. [22] studied cross-scale multi-layer metal lattice structures with different three-dimensional geometric features. At the macro scale, these structures achieve high tensile elasticity and almost constant specific strength that brittle metals do not possess. Moreover, the gradient design of a lattice structure is achieved by adjusting the density of different regions or the topological structure of individual units, exhibiting good mechanical properties and impact energy absorption characteristics. Liu et al. [16] proposed three gradient modes with characteristics of gyroscopes and diamond units, including density gradient, heterostructure gradient, and lattice unit size gradient, proving that Three Period Minimum Surface (TPMS) is an effective method for achieving multi-mode gradients. Sing et al. [17] designed cubic and honeycomb lattices with different truss diameters and densities. Research shows that the platform stress and specific energy absorption of lattices under gradient design are 67% and 72% higher than those of uniformly distributed lattices, respectively. Li et al. [15] prepared a gradient-designed Ti-6Al-4V lattice using EBM, which significantly improved the strength and energy absorption performance of the metal honeycomb lattice. Deshpande et al. [23] designed a sandwich beam with minimum weight for a given structural load index through three-point bending tests and theoretical derivation.

Existing research mainly focuses on using variable thickness design, layered design, and hybrid design to improve the mechanical properties of lattice structures. However, due to the diversity of cell types and arrangement combinations, the deformation mechanisms and mechanical properties of other novel multi-phase lattice structures are still unclear.

This article investigates the mechanical properties and energy absorption characteristics of a novel lattice structure composed of hard and soft phases under quasi-static compression. A dual-phase lattice structure was prepared using additive manufacturing technology. On this basis, experiments and numerical simulations were conducted on the dual-phase lattice structure, analyzing the deformation process, load-displacement response, bending modulus, and energy absorption performance of different types of dual-phase lattice structures. Moreover, this study explored the influence of rod diameter on load response, providing theoretical support and practical guidance for the design and optimization of metamaterials.

## 2. Specimen Preparation

**Figure 1:**
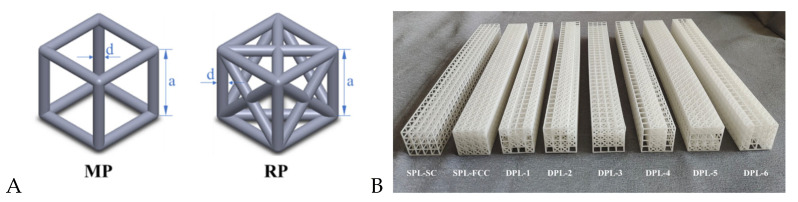
(**A**) Illustrations of the simple cube (SC) unit and the face-centered cube (FCC) unit. (**B**) The specimens of SPLs and DPLs fabricated by selective laser sintering.

As shown in Figure 1A, the dual-phase lattice (DPL) structure consists of simple cubic (SC) units as the basic phase (BP) and face-centered cubic (FCC) units as the reinforcing phase (RP). The cross-section adopts 4 × 4 units, and the ratio of the number of matrix phase to reinforcement phase cells was set to 1:1 while maintaining the symmetry of the lattice structure. A total of 34 layers of cross-sectional units were arranged along the axial array to form an array structure beam. This article presents six types of biphasic lattice beam structures with identical geometric dimensions, named DPL 1-DPL 6. The diameter d of the column that constitutes the lattice unit is 1.5 mm, the side length a of the lattice unit is 8.0 mm, and the cross-sectional side length b of the beam-type lattice structure is 32 mm, with a total length of 272 mm. The actual structural models of DPL-3 and DPL-4, as well as DPL-5 and DPL-6, are the same but differ based on different vertical contact surfaces in the three-point bending load direction. In addition, two single-phase lattice structures were designed, namely the basic phase lattice structure formed solely by a simple cubic cell array (SPL-SC) and the enhanced phase lattice structure formed solely by a face-centered cubic cell array (SPL-FCC), as the control group. The size of the single-phase lattice structure beam formed is consistent with that of the dual-phase lattice structure beam mentioned above.

The designed single and dual-phase lattice structure beams are manufactured using additive manufacturing technology [24,25,26]. Using nylon powder as the matrix material, selective laser sintering (SLS) was carried out using a FARSON 401 printer. The experimental specimens prepared are shown in Figure 1B, and the comparison between the design quality of the specimens and the actual printing quality is shown in Table 1. To facilitate understanding, the finite element model introduction uses different colors to depict the combination of the base phase and the reinforcing phase in various specimens. The printing quality error of the specimens does not exceed 10%, and the additive manufacturing process shows good stability.

## 3. Experimental Setup

**Figure 2:**
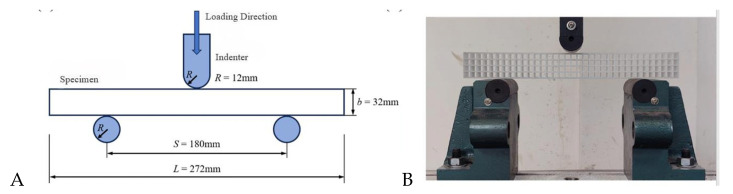
(**A**) Schematic diagram and (**B**) apparatus of quasi-static three-point bending experiment for DPLs and SPLs.

The experimental setup for the quasi-static bending loading test is shown in Figure 2. The radius R of the cylindrical indenter and supporting cylinder is 12 mm, and the span S is 180 mm. Two repeated experiments were conducted on each group of specimens. The experiment was conducted on a universal material testing machine with a range of 100 KN and a loading speed of 10 mm/min.

The mechanical properties of the nylon material used in the experiment were obtained from a study by Huang et al. [27], who conducted uniaxial tensile tests on nylon dog bone specimens using a universal material testing machine (MTS standard C 45.105), with a maximum load capacity of 100 kN. The mechanical properties of the obtained nylon material are as follows: density is 1.01 g/cm^3^, Young’s modulus is 0.6 GPa, and Poisson’s ratio is 0.3. The obtained material properties were used for material definition in finite element analysis.

## 4. Finite Element Modeling

**Figure 3:**
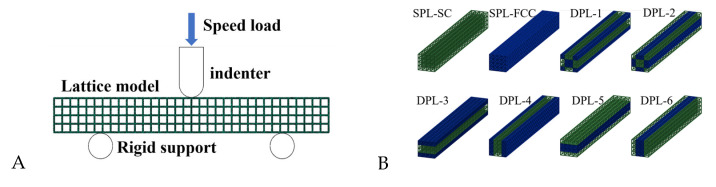

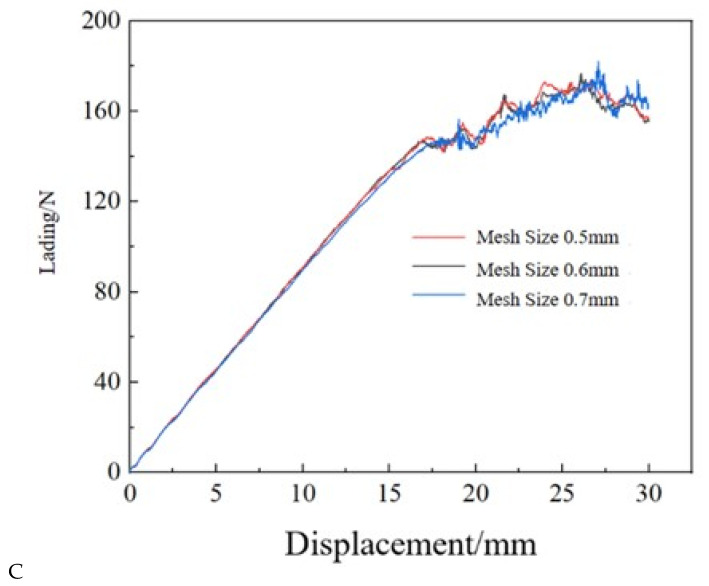
(**A**) Simulation models. (**B**) Finite element model for lateral indentation tests of SPLs and DPLs. (**C**) The load–displacement curves of SPLs under different mesh sizes.

The nonlinear finite element software ABAQUS/Explicit (ABAQUS, Inc., Rising Sun Mills, Providence, RI, USA) was used to simulate quasi-static three-point bending experiments of single- and dual-phase lattice structure beams. All test samples were simulated and analyzed, and representative simulation models are shown in Figure 3A. Figure 3B presents the finite element model for lateral indentation tests of SPLs and DPLs, which facilitates the understanding of the dual-phase structure design, with the base phase (SC) represented in green and the reinforcing phase (FCC) in blue. The quadrilateral rigid shell element (R3D4) was used to model the pressure head and support, while the beam-type lattice structure was simulated using tetrahedral solid elements (C3D4). An elastic–plastic model was used to simulate the stress–strain constitutive relationship of the matrix material. In addition, universal contact was used to define the interaction between specimens and indenters, and specimens and supports. Two supports were set with completely fixed boundary conditions, and the pressure head was set with velocity boundary conditions. For quasi-static simulation, it is necessary to increase the loading speed to complete the calculation in a reasonable time. According to the criterion proposed by Santosa et al. [28], the loading speed increases linearly to 1 m/s within the first 10 ms and remains constant throughout the subsequent process. The stress cloud map of the lattice model and the reaction and displacement data of the indenter in the result file were extracted to generate the load–displacement curve for analysis. Mesh sensitivity studies were performed on single-phase lattice structures to select the appropriate mesh sizes. The finite element model was set with three different mesh sizes: 0.5, 0.6, and 0.7 mm. The load–displacement curves are shown in Figure 3C. The results indicate that a grid with a size of 0.6 mm can ensure sufficient computational accuracy. Therefore, the unit size of the numerical analysis model was set to 0.6 mm.

## 5. Experimental and Numerical Simulation Results

This section analyzes the mechanical properties of single-phase lattice structures and dual-phase lattice structures under three-point bending load experiments and numerical simulations, including deformation modes and load responses. Through experiments and numerical simulations, the bending modulus and energy absorption effects during the elastic–plastic stage of dual-phase grid structures with different reinforcement phase arrangements were studied. Research has shown that the contact of reinforced phase elements in the vertical load direction significantly improves the energy absorption performance of the structure.

### 5.1. Load Response and Deformation Process

**Figure 4:**
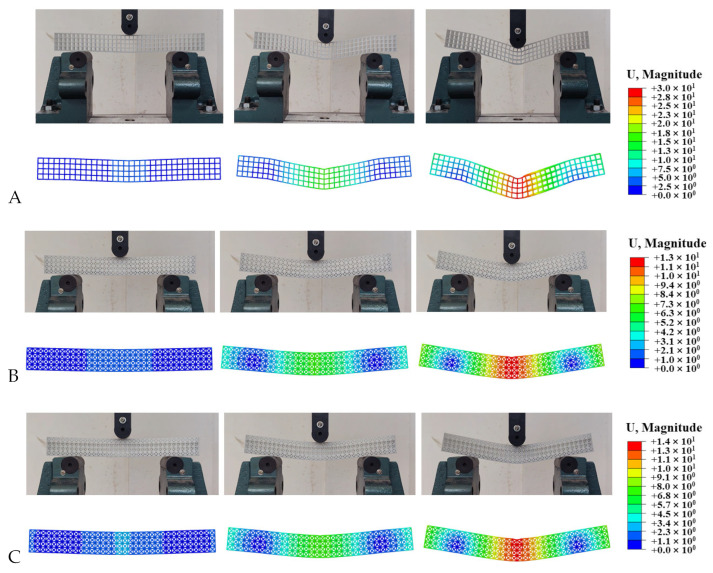

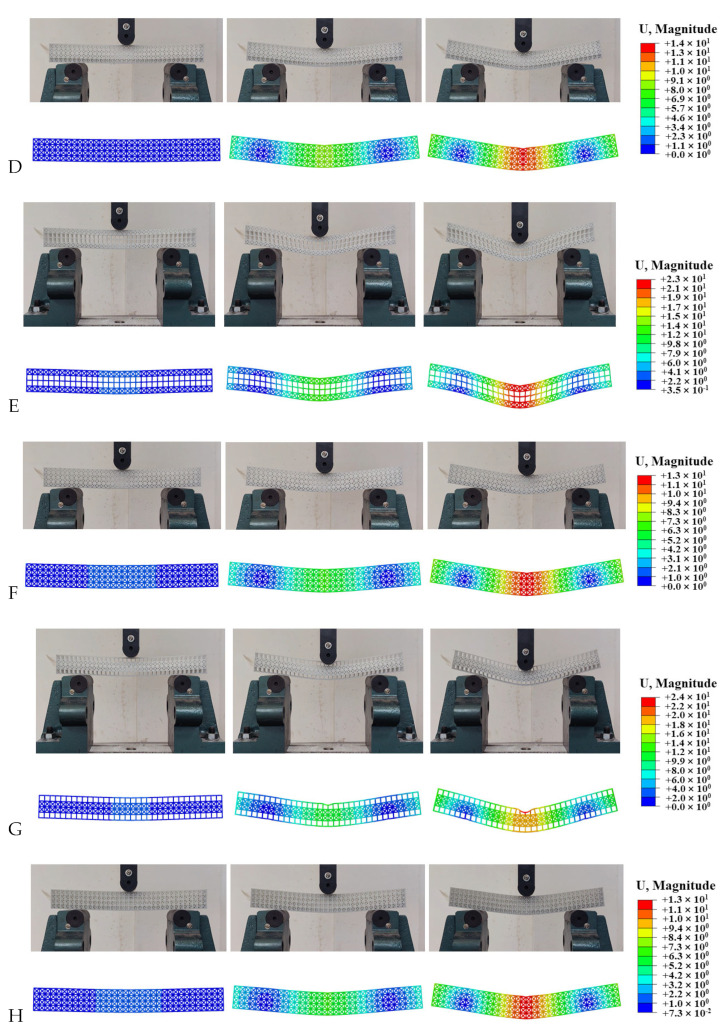
The deformation process of SPLs and DPLs under lateral loading experiments and numerical simulations. (**A**) SPL-SC beam. (**B**) SPL-FCC beam. (**C**) DPL-1 beam. (**D**) DPL-2 beam. (**E**) DPL-3 beam. (**F**) DPL-4 beam. (**G**) DPL-5 beam. (**H**) DPL-6 beam.

In this quasi-static loading test, a camera was used to record the deformation process of the sample. In repeated experiments, the samples exhibited almost identical deformation characteristics. Figure 4A shows the deformation process of the SPL-SC beam, which does not contain any reinforcing phase elements. With the displacement of the pressure head, under concentrated stress, the structure begins to sink downwards from the middle, and the beam structure bends as a whole along the middle. In addition, small bending occurs simultaneously at the support, and the entire structure is folded into four parts. The upper element at the contact point between the structure and the pressure head undergoes local deformation, and the longitudinal truss no longer maintains its original linear shape due to unstable buckling. The horizontal truss subsequently undergoes twisting deformation and lateral displacement. The unloaded SPL-SC structure exhibits partial rebound. However, due to plastic deformation and local buckling caused by previous stresses, it cannot be fully restored. Therefore, clear and visible buckling marks are still left at the contact point between the pressure head and the support position.

The deformation process of single-phase and dual-phase lattice structures with five different structural forms, SPL-FCC, DPL-1, DPL-2, DPL-4, and DPL-6, is highly consistent, as shown in Figure 4B–F. The structural characteristics of these five structures are that each of the four layers of elements in the load direction has an enhanced phase element distribution, and the enhanced phase cells are interconnected within the beam cross-section. The apparent flexure and shear of lattice beams are attributed primarily to the stretch and flexure of the crystal lattice, respectively [29]. When these lattice beam structures are subjected to three-point bending, the resulting deformation is due to the combined effects of shear forces and bending moments. In the initial stage, the contact force at the beam’s center is small, with the deformation primarily governed by the bending moment and smaller shear stress. This causes a uniform stress distribution in the middle region of the span, as shown in the second column of Figure 4. As the indenter continues to apply load, the contact force increases, leading to a localized increase in shear stress at the area near the indenter, resulting in significant shear deformation, which manifests macroscopically as a localized indentation. Compared with the SPL-SC single-phase lattice structure, these five lattice structures also exhibit local indentation at the contact position with the indenter. Obviously, the interconnection of enhanced phase units enhances the overall stiffness of the structure. To prevent shear deformation, higher stiffness is required. Distributing the reinforcing phase in the interface region where the load is applied can effectively reduce shear deformation. Conversely, placing the base phase in the interface region where the load is applied can increase the impact of shear deformation. Therefore, strengthening the distribution and connection of phases has a positive effect on preventing large-scale local buckling.

It should be noted that as a representative of these five structures, the SPL-FCC single-phase lattice structure achieved significant rebound after unloading, and the structural shape approached the original state. Compared with the obvious buckling and indentation left after unloading the SPL-SC lattice structure, the deformation of this type of lattice structure has been restored to a greater extent, leaving only slight traces.

**Figure 5:**
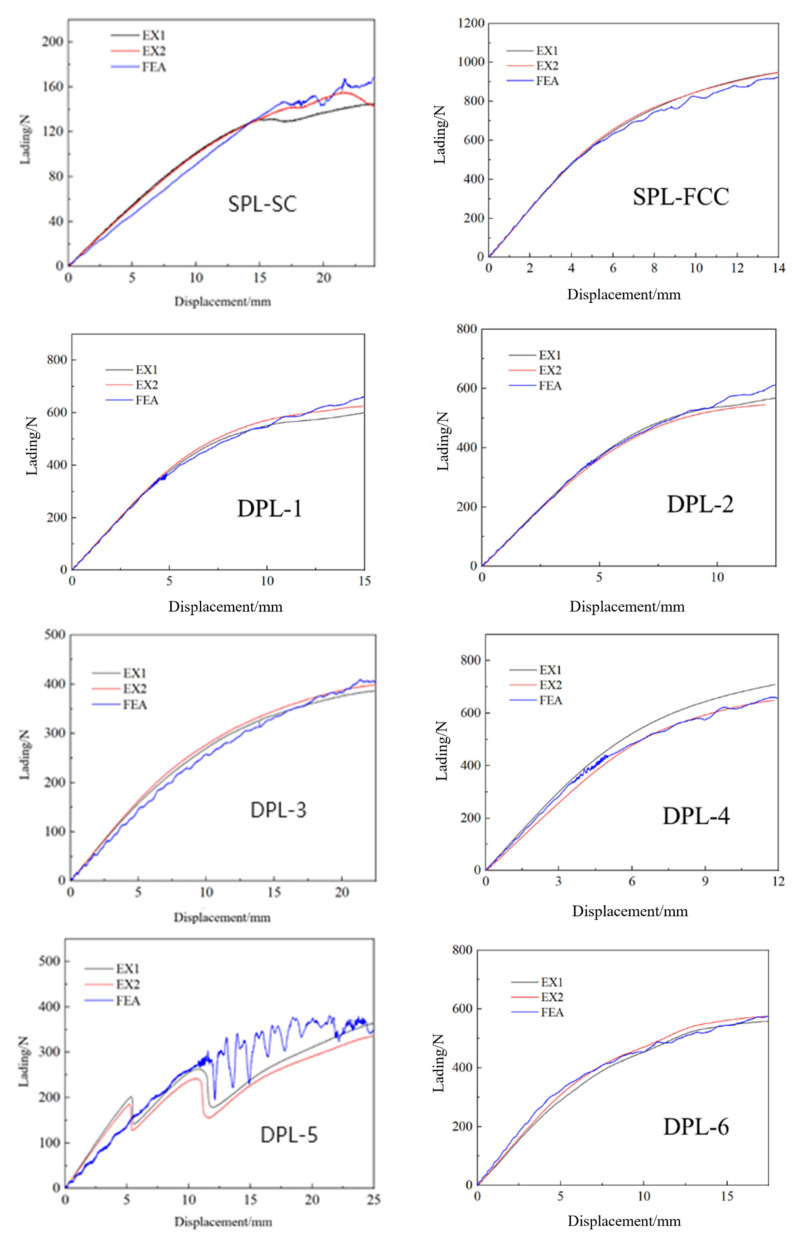
The load–displacement curves of SPLs and DPLs under three-point bending.

The three-point bending load–displacement curves of five lattice structures are shown in Figure 5. Corresponding to the deformation process, the load–displacement response laws of these five structures exhibit a high degree of consistency. After the load linearly increases in the early elastic stage, as the displacement of the pressure head gradually increases, the structure begins to enter the plastic deformation stage, and the growth rate of the load gradually slows down, becoming a nonlinear curve reflecting plastic deformation. Compared to SPL-SC lattice structures, although these structures also undergo a deformation process from elastic to plastic as the load increases, their load variation trend is smoother, without significant fluctuations. This indirectly confirms that the distribution and interconnection of enhanced phase cells layer by layer within the structure can effectively avoid local excessive buckling when subjected to local loads.

**Figure 6:**
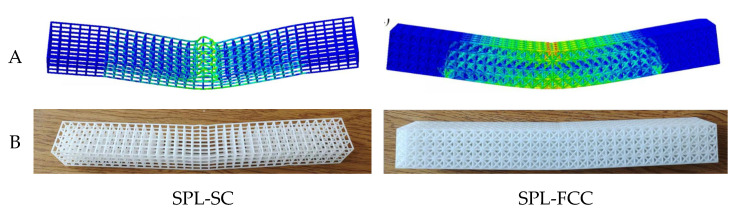
The deformation mode of SPLs under three-point bending: (**A**) simulation results; (**B**) unloaded specimen after testing.

The deformation process of the DPL-3 biphasic lattice structure is shown in Figure 4G, where the structure undergoes bending in the middle and the matrix phase cells in the middle tilt, while the deformation of the reinforcing phase cells is relatively small. Due to the reinforced phase cells being in contact with the indenter and support, the deformation is mainly borne by the matrix phase cells, and there is no significant local buckling deformation in the lattice structure. Thanks to the arrangement of basic phase cells in the middle, the deformation mode of the DPL-3 structure has more flexible characteristics. After pressure relief, the structure also rebounded, as shown in Figure 6.

DPL-3 is a typical sandwich structure with reinforcement phases arranged in the upper and lower layers, and basic phase cells distributed in the middle two layers. The corresponding three-point bending load–displacement curve of the structure is shown in Figure 5. Due to the absence of local buckling failure in the structure, the curve of the DPL-3 biphasic lattice structure follows the same trend as the curve of the second type of lattice structure, without significant fluctuations, and is also a smooth transition from elastic segment to plastic deformation.

Contrary to the DPL-3 biphasic lattice structure, the characteristic of the DPL-5 biphasic lattice structure is that the matrix phase units are distributed in the upper and lower layers, and the enhancement phase units are distributed in the middle two layers. The deformation process is shown in Figure 4H, and the overall deformation of the structure is the same as that of other lattice structures. The difference is that due to the support effect of the reinforced phase lattice cells in the middle, the stress concentration phenomenon is more obvious. The contact pressure between the upper and lower layers of the more flexible matrix phase cells and the support position undergoes more significant buckling deformation compared to the SPL-SC single-phase lattice structure. The deformation mode is shown in Figure 6, where the structure rebounds after unloading, but the local buckling indentation still exists.

The three-point bending load–displacement curve of DPL-5 is shown in Figure 5, and the overall trend of the curve remains upward. However, due to significant buckling deformation of the matrix phase lattice in contact with the indenter and support, the load curve shows significant fluctuations. There are two obvious fluctuations in the experimental curve. The first one is that the matrix phase longitudinal truss corresponding to the center of the indenter undergoes unstable buckling under axial load. As the indenter moves, it gradually transfers to the adjacent two columns of longitudinal trusses. Then, the load gradually increases, and the same unstable buckling occurs, resulting in a significant fluctuation in the second load. During the numerical simulation process, there is no clear sequence of local buckling at the contact area, so the range of curve fluctuations is more concentrated and the frequency is higher.

Based on the deformation process and the load–displacement curve obtained from the quasi-static three-point bending experiments and numerical analysis of all structures, the deformation process of additive manufacturing of the dual-phase lattice structure and its constituent single-phase lattice structure was divided into two parts: elastic and plastic. The structure undergoes overall bending along the pressure head position and rebounds after unloading. In addition, local buckling deformation occurs in the contact area between the lattice structure and the pressure head and support, and there is a significant difference in buckling deformation due to the different bending stiffness of the reinforcement phase and the matrix phase. Especially when the matrix phase elements are distributed separately in the transverse layer, the structure exhibits more flexible characteristics, which is reflected in the deformation process of the SPL-SC, DPL-3, and DPL-5 lattices. When the reinforcing phase is distributed in each layer of the unit, due to the integrity generated by the interconnection of the reinforcing phase cells, the structure exhibits rigid characteristics and the indentation area is relatively small.

### 5.2. Bending Modulus

**Figure 7:**
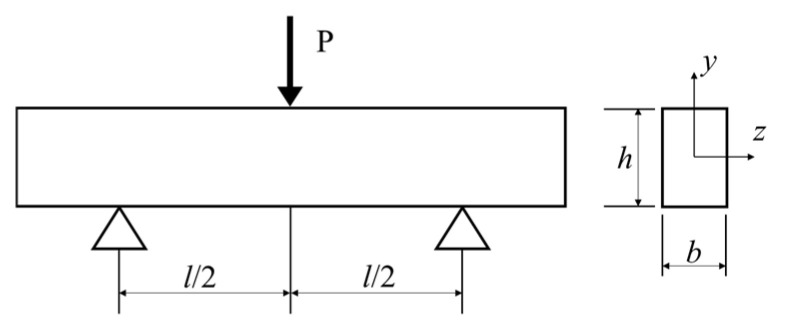
Three-point bending mechanical model.

In order to explore the ability of DPL and SPL materials to resist bending deformation within the elastic limit, it is necessary to introduce the bending modulus. The bending modulus reflects the proportional relationship between the bending stress per unit area of the material and the unit deflection it causes when an object is in a pure bending state. It can be calculated using a simplified mechanical model as shown in Figure 5, and the calculation formula is as follows:

(1) $$E_{b}=\frac{l^{3}}{48I_{Z}}\times \left(\frac{\Delta P}{\Delta f}\right)=\frac{l^{3}}{4bh^{3}}\times \left(\frac{\Delta P}{\Delta f}\right)$$

where $E_{b}$ is the bending modulus and $\frac{\Delta P}{\Delta f}$ is the slope of the elastic section of the load–deflection curve. When the deformation is small, in order to simplify the analysis, as shown in Figure 7, the sensor displacement can be approximated as the deflection of the specimen f. The formula for calculating the bending modulus is as follows:

(2) $$E_{b}=\frac{S^{3}}{4b^{4}}\times \left(\frac{\Delta P}{\Delta f}\right)$$

According to Equation (2), the bending modulus of all single and dual-phase lattice beam structures obtained from experiments and numerical simulations is calculated and compared. As shown in Table 2, the error between the numerical simulation results and the experiment is small, except for the DPL-5 lattice structure, which is within 20%.

In Table 2, it can be found that the bending modulus of the matrix-phase single-phase structure is the smallest, while the bending modulus of the reinforcement phase single-phase structure is the largest. In addition, the dual-phase design of the lattice structure is significantly better than the matrix-phase single-phase lattice structure. The bending modulus of the DPL-4 dual-phase lattice structure is the largest among the dual-phase lattice structures, which is 744.7% higher than that of the matrix-phase single-phase structure. It is worth mentioning that the enhanced phase cell contact mode of the DPL-1 and DPL-2 structures is the same, so the bending modulus is also similar. The structures of DPL-3 and DPL-4, as well as DPL-5 and DPL-6, are completely identical. However, due to the different three-point bending loading directions, the difference in bending moduli is very significant. It can be seen that the distribution of enhanced phase cells in the loading direction has a significant impact on the mechanical properties of the biphasic lattice structure.

### 5.3. Energy Absorption Characteristics

In order to investigate the energy absorption characteristics of DPL and SPL structures before failure, a deformation displacement of 0–10 mm was selected, and the energy absorbed before failure was calculated using the following formula:

(3) $$EA=\int_{0}^{\delta }F(x)dx$$

where F(x) is the instantaneous load during the deformation process.

The specific energy absorption (SEA) is the energy absorption per unit mass, which is used to describe the energy absorption efficiency of a structure. The calculation formula is as follows:

(4) $$SEA=\frac{EA}{m}$$

The energy absorption index calculations of DPL and SPL structures in numerical simulation and quasi-static loading experiments are shown in Table 3. It should be noted that the calculated absorbed energy (EA) and specific energy absorption (SEA) in the table do not consider structural failure. These values are only for comparison between different structures and do not represent the full energy absorption capacity of the biphasic lattice structure. Based on simulation and experimental data, it can be concluded that the numerical simulation results are closer to the experimental results than the bending modulus calculation results.

**Figure 8:**
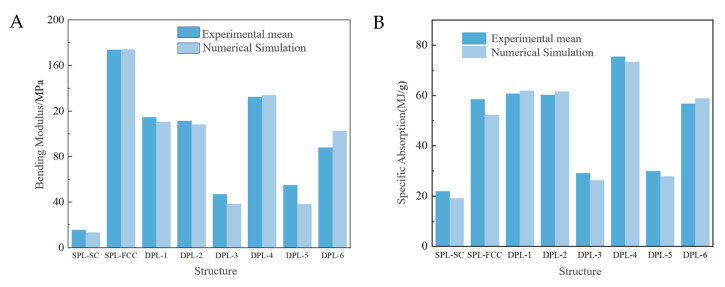
Comparison of bending moduli of single-phase and dual-phase point array structures. (**A**) Experimental and numerical results of bending moduli. (**B**) Experimental and numerical results of specific absorption.

In order to more intuitively compare the specific energy absorption of different single and biphasic lattice structures, the calculated results were plotted in a bar graph, as shown in Figure 8. The results show that the biphasic design had a more significant improvement in energy absorption indicators, and the DPL-4 biphasic lattice structure had the best performance in energy absorption compared to the matrix-phase single-phase lattice structure, with an increase of 243.5%. In addition, both the DPL-1 and DPL-2 biphasic lattice structures are slightly superior to the SPL-FCC single-phase lattice structure. Based on the specific energy absorption of the biphasic lattice, it can be inferred that its energy absorption characteristics are still highly correlated with the distribution of the loading direction of the enhanced phase units, which is reflected in the similar specific energy absorption indicators of DPL-1 and DPL-2, as well as the significant differences between DPL-3 and DPL-4, and DPL-5 and DPL-6. Comparing the specific energy absorption indices of DPL-4 and DPL-6, it can be seen that the contact between the enhanced phase units in the vertical load direction significantly improves the energy absorption performance of the biphasic lattice structure. This mechanism is also applicable to the bending modulus, but its improvement effect is limited. When the proportion of the enhanced phase units further increases, the improvement in absorption capacity is smaller than the increase in mass. Therefore, the specific energy absorption of the enhanced phase single-phase lattice structure is smaller than that of some biphasic lattice structures.

## 6. The Effect of Rod Diameter

Generally speaking, for lattice structures, the higher the relative density, the superior their mechanical properties. However, the research results of Huang et al. indicate that under axial loading conditions, the increase in relative density may lead to a decrease in the specific energy absorption index for biphasic lattice structures. To verify whether the energy absorption of biphasic lattice structure beam structures under lateral loading has the same characteristics, this section adopts the same parameterized analysis method to study the influence of relative density on the mechanical properties of biphasic lattice structures.

In this section, DPL-1 is selected as the representative of the biphasic lattice structure, and its three-point bending quasi-static simulation model is well-matched with experimental results. Similarly, by adjusting the relative density by changing the diameter of the truss rods, a three-point bending model of the DPL-1 dual-phase lattice beam structure with truss rod diameters (D) of 0.9 mm, 1.2 mm, 1.8 mm, 2.1 mm, and 2.4 mm was established based on the original model’s 1.5 mm rod diameter. All other parameters were kept unchanged, and the bending modulus (BM) and energy absorption characteristics of the dual-phase lattice structures with different rod diameters were evaluated.

**Figure 9:**
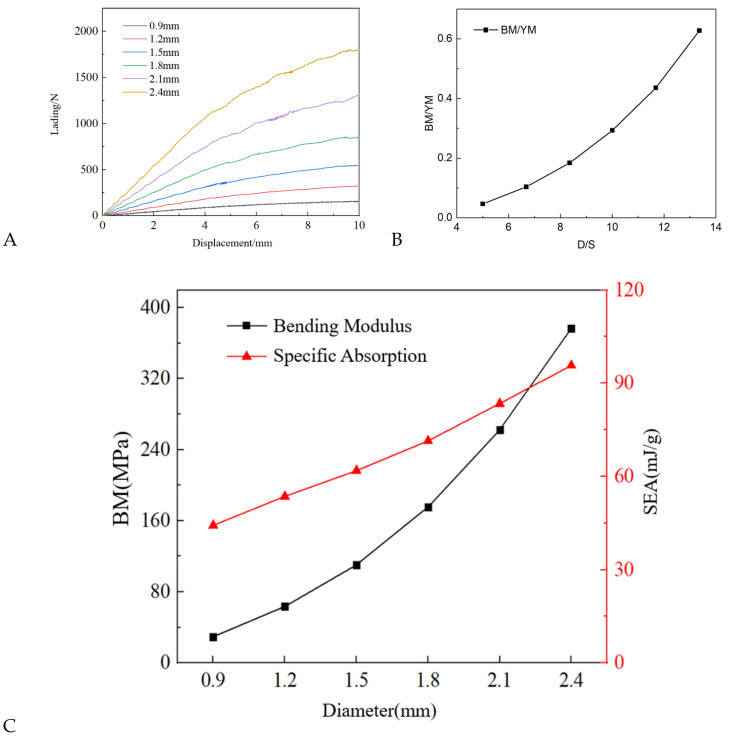
(**A**) Load–displacement curves of DPL-1 under three-point bending with different bar diameters. (**B**) Dimensionless analysis diagram of modulus and structure size. (**C**) Variation in flexural modulus and specific energy absorption of DPLs with different bar diameters.

The three-point bending load–displacement curve of the DPL-1 biphasic lattice under different pole diameters is shown in Figure 9A. According to the curve, it can be seen that the load–displacement curve trend of structures with different rod diameters under the three-point bending load is consistent, and there is no significant difference in the deformation process. The deformation process under different rod diameters is no longer shown here, and the load level gradually increases with the increase in truss rod diameter. The ratios of rod diameter to beam length and the bending modulus of the beam structure to the modulus of the base material were calculated and are presented in Table 4. Figure 9B demonstrates that increasing the ratio of rod diameter to beam length allows for the design of beam structures with a higher bending modulus using the same base material.

The bending modulus and specific energy absorption of the DPL-1 biphasic lattice structure with different rod diameters calculated based on the load–displacement curve are shown in Table 4, and the comparison between bending modulus and specific energy absorption is shown in Figure 9C. As the diameter of the rod increases, the bending modulus of the dual-phase lattice structure gradually increases, and the growth amplitude gradually increases. This means that increasing the relative density is an effective method to improve the bending stiffness of the lattice structure. In contrast, the improvement in pole diameter compared to the improvement in energy absorption performance is not so significant. As shown in Figure 8, the improvement in specific energy absorption is almost proportional to the increase in pole diameter. However, under the action of three-point bending, the biphasic lattice structure did not exhibit the phenomenon of specific energy absorption decreasing instead of increasing with the increase in truss pole diameter under compressive load. The reasons for this are twofold: firstly, the limited improvement in quality caused by an increase in relative density; secondly, the lack of a concept of densification strain under compressive load when compared through the same deformation displacement under bending load. An increase in relative density under compressive load will lead to a decrease in densification strain, thereby limiting energy absorption.

## 7. Conclusions

In this work, a dual-phase lattice structure and its composition single-phase lattice structure were studied using the three-point bending mechanism and mechanical properties under lateral loads of additive manufacturing. DPLs and SPLs were prepared using selective laser sintering technology, and numerical simulation and quasi-static loading experiments were conducted. Based on the experimental and numerical simulation results, the following conclusions were drawn:

(1) In quasi-static three-point bending experiments, both single and dual-phase lattice structures undergo overall bending deformation, and local rod buckling occurs in the contact part with the indenter. The magnitude of buckling is related to the type of contact cell, with the matrix phase cell showing more flexible characteristics.
(2) The bending resistance and energy absorption level of the matrix-phase single-phase lattice structure are relatively low. The introduction of reinforced phase cell elements in the dual-phase lattice structure can achieve a significant improvement in mechanical properties, with a maximum increase in bending modulus of 744.7% and a maximum increase in specific energy absorption of 243.5%. In addition, the specific energy absorption of some biphasic lattice structures even exceeds that of enhanced phase lattice structures, which means that the combination of soft and hard lattice designs can better achieve energy absorption under lateral loads.
(3) The results of numerical analysis and calculation are in good agreement with the experimental results in terms of deformation mode and load–displacement curve, and the calculated specific energy absorption index has an error of less than 15% compared to the experimental results. According to the numerical analysis results, it can be concluded that increasing the relative density is an effective way to improve the bending modulus and specific energy absorption of biphasic lattice structures. With the increase in rod diameter, the improvement in bending modulus is more significant than that of specific energy absorption.

## Figures and Tables

**Table 1:** Comparison of design quality and printing quality of SPLs and DPLs.

Specimen	Design Mass (g)	Printing Mass (g)		Mean (g)	Error (%)
SPL-SC	24.14	24.48	24.28	24.38	1.0
SPL-FCC	96.43	85.68	91.65	88.67	8.0
DPL-1	54.15	57.89	56.30	57.10	5.4
DPL-2	54.15	55.21	55.22	55.22	2.0
DPL-3	52.50	53.45	51.91	52.68	0.3
DPL-4					
DPL-5	49.16	48.75	47.78	48.27	1.8
DPL-6					

**Table 2:** Bending modulus of SPLs and DPLs: experiment and numerical simulation.

Specimen	Flexural Modulus (MPa)				Error (%)
	FEA	EX-1	EX-2	Mean	
SPL-SC	13.45	15.34	15.98	15.67	14.17
SPL-FCC	174.10	173.22	174.66	173.94	0.09
DPL-1	110.58	113.84	115.24	114.54	3.46
DPL-2	108.23	112.17	110.76	111.47	2.91
DPL-3	38.40	46.52	47.60	47.06	18.40
DPL-4	134.12	144.18	120.54	132.36	1.33
DPL-5	38.17	56.80	53.31	55.06	30.68
DPL-6	102.50	86.14	89.68	87.91	16.60

**Table 3:** Energy absorption indices of SPLs and DPLs: experiment and numerical simulation.

Specimen	EA (J)			SEA (mJ/g)				Error of SEA (%)
	FEA	EX-1	EX-2	FEA	EX-1	EX-2	Mean	
SPL-SC	0.46	0.53	0.54	19.06	21.74	22.15	21.94	13.13
SPL-FCC	5.04	5.16	5.20	52.27	58.19	58.64	58.42	10.53
DPL-1	3.35	3.42	3.51	61.87	59.89	61.47	60.68	1.96
DPL-2	3.33	3.37	3.28	61.50	61.03	59.40	60.21	2.14
DPL-3	1.38	1.50	1.56	26.29	28.47	29.61	29.04	9.47
DPL-4	3.85	4.18	3.76	73.33	79.35	71.37	75.36	2.69
DPL-5	1.37	1.50	1.39	27.87	31.08	28.80	29.94	6.91
DPL-6	2.89	2.67	2.81	58.79	55.31	58.21	56.76	3.58

**Table 4:** Flexural modulus and specific energy absorption of DPL-1 with different bar diameters.

D (mm)	D/S*1000	BM (MPa)	BM/YM	EA (J)	Mass (g)	SEA (mJ/g)
0.9	5.00	29.55	0.05	0.95	21.56	44.25
1.2	6.68	63.71	0.10	1.96	36.49	53.59
1.5	8.36	110.58	0.18	3.35	54.15	61.87
1.8	10.00	175.67	0.29	5.27	73.84	71.43
2.1	11.68	262.70	0.44	7.92	94.87	83.45
2.4	13.36	376.81	0.63	11.16	116.56	95.73

## Data Availability

All data included in this study are available upon request by contact with the corresponding author.
